# Potential Functions of IGFBP-2 for Ovarian Folliculogenesis and Steroidogenesis

**DOI:** 10.3389/fendo.2018.00119

**Published:** 2018-04-13

**Authors:** Marion Spitschak, Andreas Hoeflich

**Affiliations:** Institute of Genome Biology, Leibniz Institute for Farm Animal Biology (FBN), Dummerstorf, Germany

**Keywords:** IGFBP-2, ovary, follicle, folliculogenesis, steroidogenesis, aromatase

## Abstract

Ovarian follicles, as transient structural and functional complexes with the oocyte and the associated cells, determine the female reproductive cycle and thus fertility. Ovarian function is subject to the strict control of hormones and growth factors and thus regulated by auto-, para-, and endocrine mechanisms but influenced also by endogenous factors. During the waves of follicular growth and development, one follicle (monoovulatory) or a limited number of them (polyovulatory) are selected under hypothalamic–gonadal control for maturation until ovulation, resulting in the fertile oocyte. Subordinate follicles inevitably enter different stages of atresia. A number of studies have observed species-specific alterations of IGFBP-2 levels during the phases of growth and development or selection and atresia of follicles. IGFBP-2 is thus probably involved in the process of follicle growth, differentiation, and degeneration. This may occur on the levels of IGF-dependent and -independent growth control but also due to the control of steroidogenesis, e.g., *via* induction of aromatase expression. In mice, IGFBP-2 delayed reproductive development most probably by IGF-independent mechanisms. Because reproductive development is closely linked to the control of life- or health-span and energy metabolism, we feel that the time is right now to resume research on the effects of IGFBP-2 in the ovarian follicular compartment.

## Introduction

**Figure 1:**
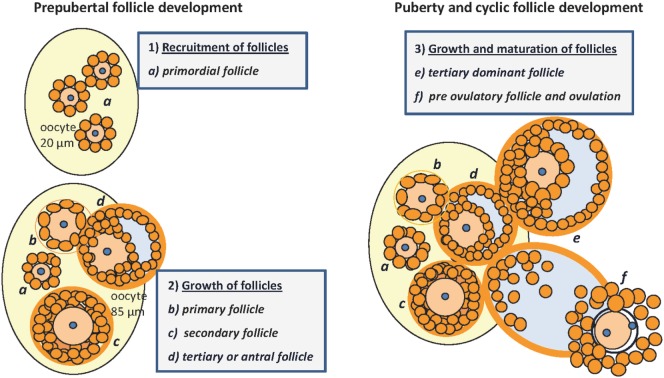
Folliculogenesis in mice: prepubertal follicular development (1) and (2): (a) pool of primordial follicles within the stroma of the neonatal ovary, the oocyte is surrounded by one layer of GC, no TC; nucleus arrested in GV I of meiosis; (b) primary follicle with one layer of cuboidal granulosa cells, signaling the recruitment of TC from the stroma; (c) secondary or preantral follicle with more than one layer of GC, recruitment of TC, and differentiation with low production of androgens; (d) tertiary follicle with formation of the antrum, proliferation of GC, maturation of TC that become steroidogenic under control of LH, increasing androgen production and E2 synthesis in GC under the control of FSH, growing to become the dominant follicle. Puberty and cyclic follicle development (3): (e) tertiary dominant follicle growth with increasing antral volume, mitosis of GC and E2 synthesis → puberty; (f) preovulatory follicle, oocyte with one layer of expanded GC and resumption of meiosis after the LH surge, increasing progesterone production → ovulation [E2, 17beta-estradiol; GC, granulosa cells; TC, thecal cells; GV, germinal vesicle; LH, luteinizing hormone; FSH, follicle-stimulating hormone; follicle classification according to (1, 4, 10, 17)].

Mammalian germ cell development is a continuous process under the strict control of hormones and growth factors that can also be affected by environmental factors. Ovarian follicles are transient functional complexes of the oocyte and associated somatic cells at different stages of development or atresia (Figure 1). Already during the prenatal phase, the proliferation and partial maturation of a species-specific number of primordial follicles take place within the stroma. By the fifth day after birth, a pool of about 8,000 oocytes within a mouse ovary (1) created in the prophase of the first meiotic maturation division (GV I) are effectively arrested under the influence of meiosis arresting factor (2, 3). However, 2 days later, the number of oocytes in mice is reduced by 60% as a result of apoptosis (1). The initiated follicle development is characterized by the appearance of a high proportion of secondary follicles around the 12th day of life (1, 4). Under the control of the hypothalamic–pituitary–gonadal axis, tertiary follicles develop with the formation of a large antrum, and their increasing 17beta-estradiol (E2) secretion finally induces the onset of puberty (5). The timing of follicular development not only depends on species or genetic background but also is under epigenetic control and can be regulated by nutrients (6, 7). However, less than 1% of primordial follicles in fact succeed to enter the cycle of follicle maturation and ovulation (8). A limited number of small tertiary follicles are responsive to increased follicle-stimulating hormone (FSH) secretion from the pituitary gland. This selection is characterized by further mitosis and maturation of the FSH-receptor expressing granulosa cells, with increasing proteoglycan synthesis, and requires the strict coordination of follicular cells and oocyte. The luteinizing hormone (LH) surge stimulates further maturation and ovulation (9). In response to LH, the resumption of meiosis is promoted by glycosaminoglycans (GAGs), secreted by granulosa cells which now also express the LH receptor and which are known to inhibit FSH (2). Oocyte maturation continues, with the production and release at ovulation of a fertilizable egg, with a haploid set of chromosomes and a separated polar body, surrounded by follicular granulosa cells. The remaining theca and granulosa cells then differentiate into the progesterone-secreting cells of the corpus luteum. Follicular atresia can be found in all stages of development, but the proportion is greatest in tertiary follicles (10). In postnatal and cyclic folliculogenesis, the total number of healthy follicles remains constant with an alternating population of secondary and tertiary follicles of between 4 and 15%, presumably due to new formation of primordial follicles (1). Puberty and cycle length or estrus intensity and duration vary line specifically and are subject to environmental factors. Ovarian function can be influenced by systemic effects, among others the body fat distribution or nutrient intake (11–13). An active contribution of IGFBP-2 or other IGFBPs for development and atresia was already postulated by Cataldo and Giudice in 1992 (14). The last 10 years have witnessed a “relative paucity” of studies on the role of IGFBPs in general (15) also including their effects on reproductive performance; the last review on the functions of IGFBPs for folliculogenesis goes back to 2002 (16), warranting an update now. The present review summarizes evidence for the interactive regulation of different ovarian developmental stages by IGFBP-2 and addresses a particular role of IGFBP-2 for the control of steroidogenesis in the maturing follicle.

## Folliculogenesis and the Control of the IGF-System

**Figure 2:**
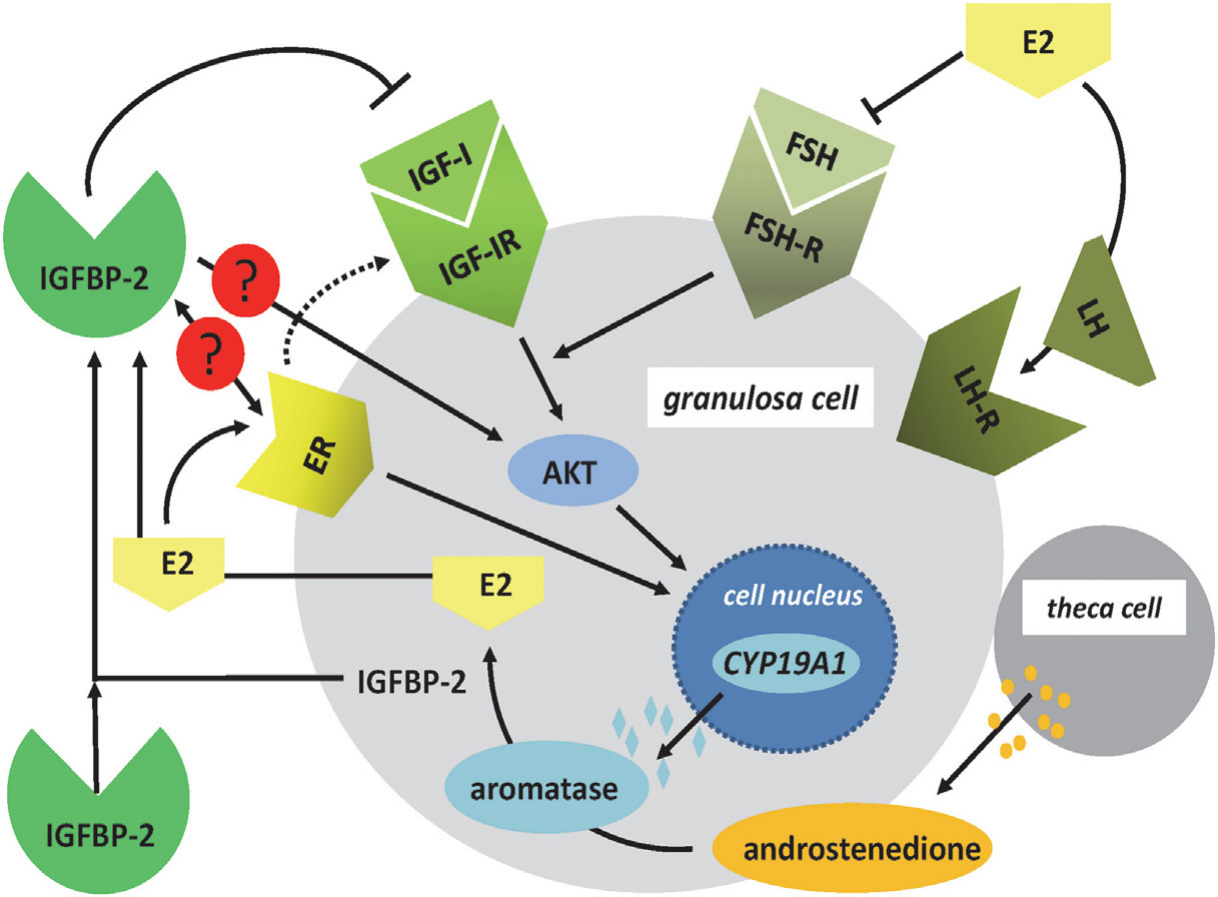
Regulation of E2 synthesis in the ovarian follicle. In granulosa cells, nuclear expression of *CYP19A1* is regulated by FSH- and ER signaling. The effects of FSH on the expression of *CYP19A1* gene are mediated by IGF-IR signaling and AKT. ER signaling can also include the IGF-IR pathways. In addition, the ER has direct effects on *CYP19A1* gene expression within the cell nucleus. Androstenedione, produced by theca cells, is used as a substrate for aromatase to produce E2. In granulosa cells, IGFBP-2 is regulated by E2, and IGFBP-2 has been shown to block FSH-dependent E2 production. Increased levels of E2 block the expression of FSH and induce the LH surge. Open questions in granulosa cells include the function of IGFBP-2 for ER- and IGF-independent effects of IGFBP-2 for E2 production (AKT, protein kinase B; *CYP19A1*: aromatase; E2, 17beta-estradiol; ER, estrogen receptor; FSH, follicle-stimulating hormone; IGF-IR, IGF-I receptor; LH, luteinizing hormone).

Insulin-like growth factor-1 (IGF-I) is produced already in granulosa cells of murine primary follicles and with a maximum in late preantral and early antral follicles, where it is associated with antrum remodeling and the growth of healthy follicles (18). In the brain, and depending on the concentrations of E2, IGF-I was demonstrated to control the hypothalamic release of LH and reproductive development in female rats (5). In human granulosa cells, IGF-I receptor signaling (19) is permissive for the positive effect of FSH on the expression of aromatase (CYP19A1) mediated by AKT signaling (Figure 2). The differentiation from the preantral to the large antral follicle requires IGF-IR activity with subsequent AKT activation for FSH-induced steroidogenic gene expression, which in turn is maintained *via* synergistic effects with local IGF-II (19, 20). Mice lacking the IGF-I receptor in ovarian granulosa cells are devoid of antral follicles, show high rates of apoptosis, low AKT activation, low levels of serum E2, and are infertile (21). Synergistically with FSH, IGF-I stimulates activation of AKT-dependent aromatase expression, cell proliferation, and expression of apoptosis-regulating genes in the granulosa cells (22–24). The positive effect of FSH on expression of aromatase was blocked by the addition of IGFBP-2, and an excess of IGF-I was able to abolish the inhibitory effect of IGFBP-2 on FSH-dependent aromatase expression (22). Accordingly, the negative effect of IGFBP-2 on steroidogenesis was IGF dependent in granulosa cells. IGF-I is also one of the stimulators of androgen synthesis in the theca cells under LH control (17). Thus, IGF-I, under the influence of gonadotropic hormones, is an essential regulatory component for the growth of antral follicles and their increasing E2 biosynthesis in humans, mice, and rats. Furthermore, IGF-I expression during folliculogenesis in mice and rats is controlled by estrogen receptors α and β. E2 has an autocrine dose-dependent stimulatory or inhibitory effect on the IGF-I-IGF-IR pathway (25–27).

## Folliculogenesis and the Control of IGFBP-2

IGFBP-2 is present in follicular fluid and subject to dynamic changes during follicle growth and maturation. Accordingly in sows, IGFBP-2 was reduced during follicular development (28). Within the mouse ovary, IGFBP-2 can be localized in discrete regions characterized by altered follicular growth, developmental stage, and atresia and thus accordingly was discussed in a functional context of folliculogenesis (18). Equine growing follicles exclusively produced IGFBP-2, and dominant follicles had lower concentrations of IGFBP-2 (29). In this experimental setting, E2 increased expression of IGFBP-2 and FSH increased expression of IGFBP-2 *in vitro* (29). During selection to a healthy dominant follicle in heifers, the capacity of the granulosa cells to enhance steroid synthesis consistently correlated with low concentrations of IGFBP-2 (30). The dominance of follicles was associated with lower amounts of IGFBP-2 and markedly higher E2 contents (30). This observation is in line with substantial increases in IGFBP-2 in follicular fluids of subordinate follicles derived also from heifers (31, 32). There were transient increases in LH-induced differentiation with enhanced IGF-I and E2, but decreased IGFBP-2 (33). Interestingly, the levels of IGFBP-2 were in a positive correlation when compared to caspase-3 activity (31, 32). IGFBP-2 expression was reduced in granulosa cells simultaneously with increased expression of IGF-1 and IGF-1R as also the steroidogenic genes responsible for synthesis from cholesterol to E2 and progesterone (34). From the dynamic changes in IGFBP-2 expression/concentration during folliculogenesis or because of the correlations of IGFBP-2 with reproductive hormones, an active contribution of IGFBP-2 during the maturation of follicles has been assumed with an effect also on the expression of aromatase in growing bovine follicles (35).

The reduction of local IGFBP-2 or other IGFBPs (36) in the follicular compartment can also be a result of active proteolysis. In dominant follicles, proteolytic degradation of IGFBP-4 and -5 and lower concentrations of IGFBP-2 were discussed in the context of increased levels of free IGF-I, and a separate review was dedicated to the control of IGFBPs during follicle selection (37). In bovine follicles, it was demonstrated that IGFBP-2 proteolytic activity originates from granulosa cells but not from the oocyte, and a self-regulatory mechanism of IGF-I activation in granulosa cells was discussed by the authors (38). For further reading on the effect of PAPP-A-dependent IGFBP-proteolysis on the selection of dominant follicles, we would like to refer to the actual discussion of Monget and Mazerbourg (39).

## Follicular Atresia and the Control of IGFBP-2

In the ovaries from polyovulatory as well as in monoovulatory females, permanent follicle selection with development and atresia is taking place. This process is subject to hypothalamo-pituitary control in interaction with intra-ovarian control. Distinct characteristics of follicular atresia are present at the level of morphology and apoptosis, but also lower E2 concentrations can indicate atretic degeneration of follicles. When compared to healthy or atretic follicles from human donors, IGFBP-2 concentrations were increased in human atretic follicular fluid (40). IGFBP-2 was also increased in atretic follicles from pigs after estrus (41). At the same time, E2 was decreased, whereas apoptosis was increased in follicles from pigs (41) and humans (40), and therefore, the authors discussed control of IGFBP-2 concentrations by E2. As reviewed before, expression of IGFBP-2 by steroids is observed in multiple tissues including various tissues from the female reproductive system (42). Notably, the vast majority of studies identified positive effects of exogenous steroids on the expression of IGFBP-2 (42). In mice, higher expression of IGFBP-2 mRNA was associated with late but not with earlier stages of atresia (18). The potential effects of IGFBP-2 on follicular atresia could be mediated by IGF-dependent or IGF-independent mechanisms (43). Interestingly, IGFBP-2 was able to inhibit FSH-dependent induction of aromatase and cholesterol side-chain cleavage enzyme (CYP11A1) expression (22). The inhibitory effect of IGFBP-2 was compensated by the addition of excess IGF-I, and the contribution of IGFBP-2 in the control of steroidogenesis thus cannot be excluded (22).

## Effects of IGFBP-2 on Reproductive Performance

In granulosa cells from polycystic follicles isolated from dairy cows, reduced mRNA expression of IGFBP-2 was found when compared to granulosa cells from normal follicles (44). Also in human granulosa cells isolated from polycystic ovaries, IGFBP-2 expression was reduced when compared to controls (45). Therefore, an active contribution of IGFBP-2 on reproductive performance might be indicated. In fact, single nucleotide polymorphisms (SNP) in the *IGFBP2* gene locus were identified as candidate markers for reproduction traits or litter size in different pig populations (46, 47). In dairy cows, reproductive development (e.g., age of first conception or calving) was correlated with a number of distinct SNPs on the *IGFBP2* gene (48). In fact, forced expression of IGFBP-2 delayed reproductive development in female transgenic mice (49). In this model, the expression of wildtype but not mutated IGFBP-2 delayed the onset of first estrus and hence ovarian cycle activity (49). Mutated IGFBP-2 lacked the integrin binding sequence and was thus discussed in a functional context in regard to altered reproductive performance; the negative effect of IGFBP-2 on reproductive development appears to be IGF independent. Regulation of IGFBP-2 expression by steroid hormones is observed in different vertebrate species in multiple cells and tissues, including the follicle (42). In addition, a mutual relationship was observed between expression of IGFBP-2 and estrogen receptors in breast epithelial cells (50). Notably, the presence of the RGD motif was also required for the effects on ER expression as demonstrated by Foulstone et al. (50). However, the relationship between estrogen receptor expression and IGFBP-2 remains to be assessed in ovarian follicles.

## Summary and Conclusion

IGFBP-2 is present in high abundance in follicular fluid and a number of studies identified IGFBP-2 by Western ligand blotting. Accordingly, it is unclear why mainly only descriptive studies are available on the functions of IGFBP-2 in regard to folliculogenesis. Studies describing altered expression of IGFBP-2 in growing versus atretic, in dominant versus subordinate, or in earlier versus later stages of follicles are available. The majority of studies reported lower levels of IGFBP-2 in healthy, larger, or later developmental stages of follicles or higher expression of IGFBP-2 in atretic follicles.

In follicles, IGFBP-2 is regulated by steroids, FSH, and LH, and there is experimental evidence that also steroidogenesis is negatively coregulated by IGFBP-2. The effect of IGFBP-2 on steroidogenic gene expression, including aromatase in the dominant follicles, could be exerted by IGF-dependent or IGF-independent mechanisms. In fact, in IGFBP-2 transgenic mice, the negative effects on reproductive development have been suggested to be IGF independent. In ovarian follicles, so far only IGF-dependent effects of IGFBP-2 on steroidogenesis have been provided. Since AKT is regulated both in an IGF-dependent and -independent fashion, e.g., by integrins or proteoglycans in various cell types and required for steroidogenesis in follicles, AKT appears as an attractive target for future research also on IGF-independent effects of IGFBP-2 during folliculogenesis and steroidogenesis.

## Author Contributions

MS and AH have written the manuscript. MS has developed the figures.

## Conflict of Interest Statement

AH is related to Ligandis UG.
